# Microarchitectural changes in the mandibles of ovariectomized rats: a systematic review and meta-analysis

**DOI:** 10.1186/s12903-019-0799-0

**Published:** 2019-06-26

**Authors:** Jeong-Hee Lee, Sang-Sun Han, Chena Lee, Young Hyun Kim, Bulgan Battulga

**Affiliations:** 1 Department of Oral and Maxillofacial Radiology, Yonsei University College of Dentistry, 50-1 Yonsei-ro Seodaemun-gu, Seoul, 03722 Korea; 2 School of Dentistry, Mongolian National University of Medical Sciences, Ulaanbaatar, Mongolia

**Keywords:** Mandible, Meta-analysis, Ovariectomy, Osteoporosis, X-ray, Microtomography

## Abstract

**Background:**

This study aimed to examine radiologic microarchitectural changes in the mandibles of ovariectomized (OVX) rats through a systematic review and meta-analysis and to identify factors of the OVX rat model that influence on the bone microstructure.

**Methods:**

Eligible articles were identified by searching electronic databases, including Embase, Medline, Web of Science, and KoreaMed, for articles published from January 1966 to November 2017. Two reviewers independently performed study selection, data extraction, and quality assessment. The pooled standardized mean difference (SMD) with 95% confidence intervals was calculated using a random-effects model. Subgroup analysis and meta-regression were performed to explore the effect of potential sources on the outcomes. The reliability of the results was assessed by sensitivity analysis and publication bias.

**Results:**

Of 1160 studies, 16 studies (120 OVX and 120 control rats) were included in the meta-analysis. Compared to the control group, the OVX rats’ trabecular bone volume fraction (SMD = − 2.41, *P* < 0.01, I^2^ = 81%), trabecular thickness (SMD = − 1.73, *P* < 0.01, I^2^ = 73%) and bone mineral density (SMD = − 0.95, *P* = 0.01, I^2^ = 71%) displayed the bone loss consistent with osteoporosis. The trabecular separation (SMD = 1.66, *P* < 0.01, I^2^ = 51%) has widen in the OVX mandibular bone in comparison to the control group. However, the trabecular number showed no indication to detect the osteoporosis (SMD = − 0.45, *P* = 0.38, I^2^ = 76%). The meta-regression indicated that longer post-OVX periods led to greater changes in bone mineral density (*β* = − 0.104, *P* = 0.017). However, the rats’ age at OVX was not linked to bone microstructure change.

**Conclusions:**

Using meta-regression and sensitivity analysis techniques, heterogeneity across the micro CT studies of OVX-induced osteoporosis was found. The major factors of heterogeneity were the region of interest and post-OVX period. Our assessment can assist in designing experiments to maximize the usefulness of OVX rat model.

## Background

Osteoporosis is a condition defined by reduced bone mass and microarchitectural deterioration of bone tissue, leading to a severe risk of deformity, pain, or skeletal fracture [1, 2]. Osteoporosis has become a major public health concern [3] which commonly occurs in postmenopausal women as a result of ovarian atrophy and the related decrease in circulating estrogens [4]. Osteoporosis is known to affect all skeletal sites, but the degree of bone loss and microarchitectural changes are not uniform [5]. Mandibular bony changes in systemic osteoporosis are a critical concern in the dental field as the success of implant osseointegration and another dental surgery is associated with the bone quality and quantity of the mandible [6, 7].

Ovariectomized (OVX) rats have been used as a preclinical model of postmenopausal humans to investigate mandibular changes [8, 9]. In OVX rats, bone mineral density (BMD) changes are similar to those in humans; therefore, BMD can be used as a common indicator of bone mass [10]. Some studies using OVX rats reported a relationship between systemic osteoporosis and mandibular bone loss [6, 11–13]; however, others have suggested a weak relationship, or no relationship at all [14–17]. The divergent results in the previous literature make it challenging to draw firm conclusions.

To find a reliable result in OVX rat studies, it is important to identify the factors that influence on bone structural changes. These animal studies can detect quite a heterogeneity in terms of species, designs, and intervention protocols compared to clinical trials [18]. In various studies of OVX rats, there are inconsistencies in the rat strain, the age of the rats, and post-OVX period. In addition, the irregular shape of the mandible hinders the establishment of criteria for observation site selection, which led to inconsistencies in the region of interest (ROI) among previous studies [19]. These factors underscore the need to establish a standard model by further investigating the characteristics of studies of OVX-induced osteoporosis. In such a context, meta-analysis can be a constructive tool to shed light on, and perhaps resolve, the lack of existing consensus.

Recently, researchers have examined trabecular morphology by using micro-computed tomography (micro-CT) to characterize bone deterioration in OVX rats [14]. Micro-CT has advantages, such as being non-destructive, fast, and easy compared to histological sections [20]. We aimed to examine radiologic microarchitectural changes in the mandibles of OVX rats through a systematic review and meta-analysis and to identify factors of the OVX rat model that influence on the bone microstructure.

## Methods

### Literature search and study selection

The systematic review and meta-analysis were conducted in accordance with Preferred Reporting Items for Systematic Review and Meta-analysis (PRISMA) statement [21]. Studies of radiologic microarchitecture in OVX rats were searched in Embase, Medline, Web of Science, and KoreaMed (published from January 1966 to November 2017) using the following keywords: rat, rats, mandible, jaw bone, alveolar bone, osteoporotic, osteoporosis, ovariectomy, ovariectomized, and postmenopausal. Two of the authors (JHL and YHK) reviewed the title and abstract of the publications found in the literature search and made a preliminary selection. The final selection of studies was independently made by the same 2 individuals, according to the inclusion and exclusion criteria presented below (Table 1). Disagreements about the selection of studies and interpretation of data were settled by a third author (CL), and consensus was reached after discussion with all authors.

**Table 1:** Literature search selection criteria

Selection criteria	1	In vivo experimental studies including ovariectomized (OVX) and control rats
	2	Used the mandible as the observation site
	3	Reported outcomes of at least 1 bone morphometric parameter with micro-CT (expressed as trabecular bone volume fraction [BV/TV], trabecular thickness [Tb.Th], trabecular number [Tb.N], and trabecular separation [Tb.Sp]), or bone mineral density (BMD).
Exclusion criteria	1	Drug-induced or elderly osteoporosis model
	2	Use of medication or treatment as the intervention for the OVX rats (exception: partial data of non-intervened subjects from related intervened and non-intervened data)
	3	Reports of non-experimental studies (reviews, letters and expert opinion publications)
	4	Publications without full text

### Data extraction

Data extraction was done independently by 2 authors (JHL and YHK). For each eligible study, we extracted data relating to study characteristics and outcomes using a pre-defined form including the name of the first author, the year of publication, the strain and number of the OVX and control rats, the rats’ age at OVX, the period after OVX surgery, the ROI of the mandible (mandibular body, mandibular condyle, M1 interradicular septum) and microarchitectural outcome (BV/TV, Tb.Th, Tb.Sp, Tb.N, BMD). Studies with more than one mandibular ROI were included independently in the meta-analysis. The mean value and standard deviation of the above data, as well as the number of OVX and control rats, were extracted for the meta-analysis. The data reported in graphs were estimated with GetData Graph Digitizer version 2.26 (Fedorov. S, 2013, Getdata-graph-digitizer.com, Russia).

### Quality assessment

The methodological quality of the individual studies was assessed by 2 authors (JHL and YHK) independently based on the Collaborative Approach to Meta-analysis and the Review of Animal Data from Experimental Studies checklists [22] and the Instruments for Assessing Risk of Bias and Other Methodological Criteria of Published Animal Studies [23]. These guidelines assess quality using the following criteria: (1) sample size calculation; (2) random allocation to treatment; (3) husbandry conditions (e.g., breeding program, light/dark cycle, temperature, type of food, access to water, and environmental enrichment); (4) blinded assessment of outcomes; (5) compliance with animal welfare regulations; (6) disclosure of conflicts of interest; and (7) peer-reviewed publication. The quality scale ranged from 0 to 7 points. A third reviewer (CL) settled any disagreements between the 2 reviewers.

### Statistical analysis

Microarchitectural changes in the mandibles of OVX rats were identified using the standardized mean difference (SMD) with 95% confidence intervals (CIs), using a random-effects model with the DerSimonian-Laird method. To explore heterogeneity among the studies, the Cochran Q statistic and Higgins’s I^2^ statistic were used for each outcome. *P*-values less than 0.1 or an I^2^ statistic greater than 50% was defined as statistically significant heterogeneity [24].

The subgroup analysis and meta-regression analysis were performed to identify factors potentially responsible for heterogeneity among the studies included in the meta-analysis. For potential sources that were categorical variables, subgroup analysis was used to compare their effect sizes. Subgroup analysis based on analysis of variance assumes between-study variance (τ^2^) to be the same in all subgroups. For potential sources that were continuous variables, meta-regression was used as an indicator of the possible influence on the effect size. The potential sources of interest were the strain of the rats, the age of the rats, the ROI of the mandible, the period after OVX, and the quality score of literature. Additionally, we performed a sensitivity analysis to assess the robustness of our findings.

The contour-enhanced funnel plot, Egger’s regression test, and Begg’s rank correlation test were applied to assess the presence of publication bias [25, 26]. When there was evidence of publication bias, potentially missing studies were imputed using the trim-and-fill method [27]. The number of missing studies was estimated using fail-safe numbers, as an additional method to assess publication bias. It has been suggested that a fail-safe number exceeding 5 K + 10 (K = N studies in the meta-analysis) should be considered acceptable [28]. Statistical analysis was performed using Review Manager (version 5.3.5 for Windows; the Cochrane Collaboration, Oxford, UK) and R software version 3.4.3 (The R Foundation for Statistical Computing; Vienna, Austria). All statistical tests were 2-sided, and *P* values < 0.05 were considered to indicate statistical significance.

## Results

### Selection of literature

**Fig. 1:**
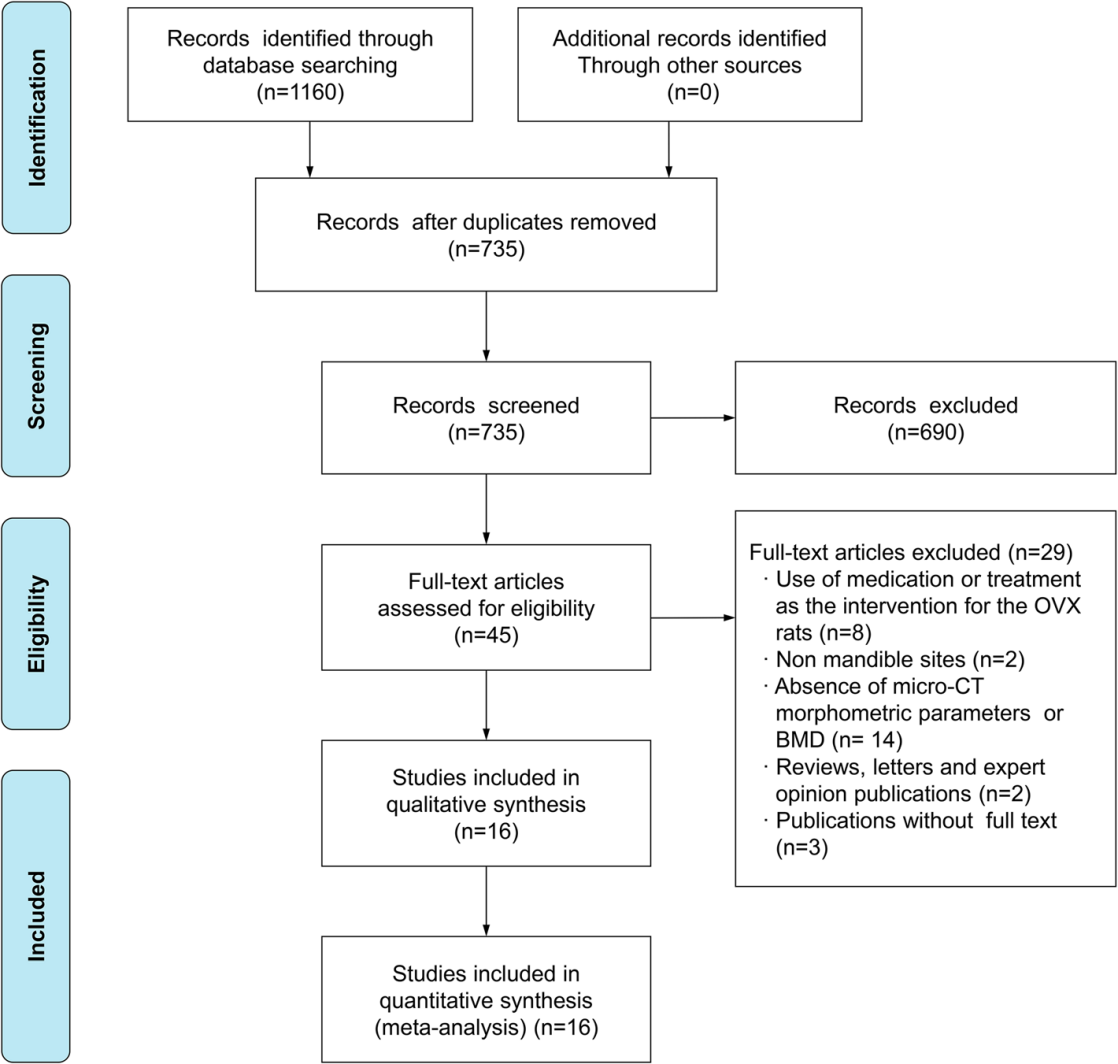
Flow diagram of literature search, and selection criteria adapted from PRISMA. OVX, ovariectomy; CT, computed tomography; BMD, bone mineral density

The literature selection strategy is shown in Fig. 1. A total of 1160 publications from electronic databases were identified through electronic and manual searches. After screening the titles and abstracts, 45 studies remained. The full text of the 45 remaining studies was thoroughly reviewed. Of those, 29 studies were excluded due to the use of medication or treatment as an intervention for the OVX rats; the assessment of sites other than the mandible; the absence of bone morphometric parameters using micro-CT or BMD measurements; being reviews, letters, or expert opinion publications; or the absence of available full-text versions. Subsequently, 16 articles were finally selected for the qualitative synthesis.

### Characteristics of the included studies

The characteristics of the eligible studies are shown in Table 2. The meta-analysis included 16 comparative assessments of post-OVX microarchitectural changes (120 rats in the OVX group and 120 rats in the control group). In each study, one particular ROI such as mandibular body or condyle was selected from multiple comparisons into the Meta-analysis (Kim KH [29] and Kuroda S [30]). One of the 16 studies was written in Korean [29], and the rest were in English.

**Table 2:** Description of the characteristics of the included studies

Study	Strain of rats	Number of OVX rats	Number of control rats	Age of rats at OVX (weeks)	Post-OVX period (weeks)	Outcome (relevant to this review)	Region of interest (ROI)
Hsu PY 2016 [6]	Wistar	6	6	8	12	BV/TV, Tb.Th, Tb.Sp Tb.N,	Mandibular body
Jiang L 2017 [31]	Sprague-Dawley	10	10	25	12	BV/TV, Tb.Th, Tb.Sp	Mandibular condyle
Kim KH 2002 [29]	Sprague-Dawley	3	3	24	1, 2, 3, 4, 6, 8, 12, 16	BMD	Mandibular body
Kuroda S 2003 [30]	Sprague-Dawley	6	6	13	15	BMD	Mandibular condyle
Li CL 2014 [12]	Sprague-Dawley	8	8	13	18	BV/TV, Tb.Th, Tb.Sp, Tb.N, BMD	M1 interradicular septum
Liu H 2015 [19]	Sprague-Dawley	10	10	NR	12	BMD	Mandibular body
Liu XL 2015 [32]	Sprague-Dawley	3	3	25	2, 4, 12, 24, 36	BV/TV, Tb.Th, Tb.Sp, Tb.N, BMD	M1 interradicular septum
Liu Z 2015 [33]	Wistar	7	7	12	21	BV/TV, Tb.Th, Tb.Sp, Tb.N, BMD	M1 interradicular septum
Liu ZS 2015 [34]	Wistar	10	10	8	12	BMD	M1 interradicular septum
Mavropoulos A 2014 [35]	Sprague-Dawley	10	10	25	16	BV/TV, Tb.Th, Tb.Sp, Tb.N	Mandibular body
Moriya Y 1998 [15]	Sprague-Dawley	5	5	4	4	BMD	Mandibular body
Patullo IM 2009 [36]	Wistar	6	6	17	9	BMD	Entire mandible
Sun W 2014 [40]	Sprague-Dawley	10	10	NR	12	BV/TV, Tb.Th, Tb.Sp, Tb.N	Mandibular body
Tanaka M 1998 [37]	Fischer	8	8	17	1, 2, 4, 8	BMD	Mandibular condyle
Tanaka M 2003 [38]	Fischer	6	6	25	51	BV/TV, Tb.Th, Tb.Sp, Tb.N	M1 interradicular septum
Yang J 2003 [39]	Lewis-Brown-Norway	12	12	12	16	BV/TV, Tb.Th, Tb.Sp	Mandibular body

*OVX* Ovariectomy, *BV/TV* Trabecular bone volume fraction, *Tb.Th* Trabecular thickness, *Tb.N* Trabecular number, *Tb. Sp* Trabecular separation, *BMD* Bone mineral density, *NR* Not reported, *M1* First molar

Different rat strains were used in each study, including Sprague-Dawley, Wistar, Fischer, and Lewis-Brown-Norway rats. The age of the rats when the OVX was performed ranged from 4 weeks to 25 weeks. Two studies did not report the age of the rats [19, 40]. The sample size of the OVX and control groups in all 16 studies ranged from 3 to 10. The post-OVX period for radiologic microarchitecture assessments was performed varied from 4 weeks to 48 weeks. Thirteen studies used 1 post-OVX period, while 3 studies had 4 or more post-OVX periods. In such cases, only the final measurements were included. Moreover, the bone morphometric parameters of trabecular microarchitecture were diverse. Data on BMD were found in 10 studies [12, 15, 19, 29, 30, 32–34, 36], trabecular bone volume fraction (BV/TV), trabecular thickness (Tb.Th), and trabecular separation (Tb.Sp) were available in 9 studies [6, 12, 31–33, 35, 38–40], and trabecular number (Tb.N) was reported in 7 studies [6, 12, 32, 33, 35, 38, 40]. The most commonly selected ROIs in the mandible was the mandibular body and the interradicular septum of the first molar (M1). The rest of the studies used the entire mandible and mandibular condyles.

### Quality assessment

The risk of bias for all 16 studies is shown in Table 3. Eight studies received a quality score of 4 or higher [6, 12, 15, 31, 34, 36, 38, 40]. The lowest quality score was 1 [19, 29], and the highest quality score was 6 [31]. No study described the sample size calculation, and 2 studies used a blinding method in outcome assessment [31, 36]. Twelve studies mentioned the random allocation of rats to groups [6, 12, 15, 19, 31–38, 40]; however, none stated the precise method of randomization. Ten studies mentioned the husbandry conditions of the rats [6, 12, 15, 29, 31, 34, 36–38], and all studies except for 4 explained their compliance with animal welfare regulations [19, 29, 37, 39]. A statement of potential conflicts of interest was only presented in 4 studies [12, 31, 35, 40].

**Table 3:** Quality assessment of the studies included in the meta-analysis

Study	Sample size calculation	Random allocation to treatment	Husbandry conditions	Blinded assessment of outcomes	Compliance with animal welfare regulations	Conflicts of interest disclosed	Peer-reviewed publication	Quality score
Hsu PY 2016 [6]	N	Y	Y	N	Y	N	Y	4
Jiang L 2017 [31]	N	Y	Y	Y	Y	Y	Y	6
Kim KH 2002 [29]	N	N	Y	N	N	N	N	1
Kuroda S 2003 [30]	N	N	N	N	Y	N	Y	2
Li CL 2014 [12]	N	Y	Y	N	Y	Y	Y	5
Liu H 2015 [19]	N	Y	N	N	N	N	N	1
Liu XL 2015 [32]	N	Y	N	N	Y	N	Y	3
Liu Z 2015 [33]	N	Y	N	N	Y	N	Y	3
Liu ZS 2015 [34]	N	Y	Y	N	Y	N	Y	4
Mavropoulos A 2014 [35]	N	N	N	N	Y	Y	Y	3
Moriya Y 1998 [15]	N	Y	Y	N	Y	N	Y	4
Patullo IM 2009 [36]	N	Y	Y	Y	Y	N	Y	5
Sun W 2014 [40]	N	Y	N	N	Y	Y	Y	4
Tanaka M 1998 [37]	N	Y	Y	N	N	N	Y	3
Tanaka M 2003 [38]	N	Y	Y	N	Y	N	Y	4
Yang J 2003 [39]	N	N	Y	N	N	N	Y	2

### Meta-analysis

#### BV/TV changes in the mandibles of OVX rats

**Fig. 2:**
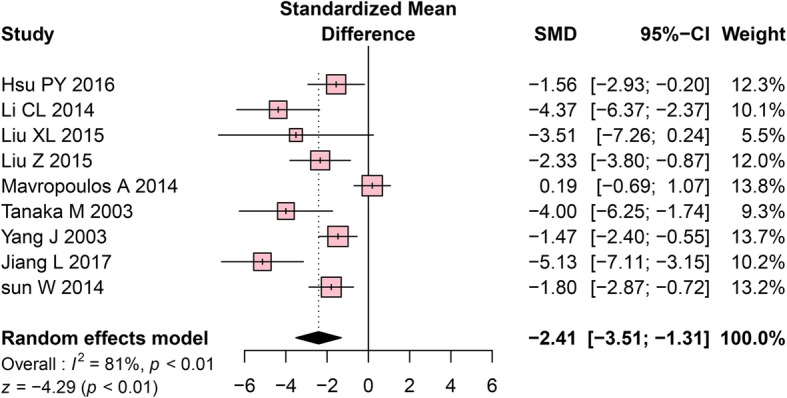
Forest plot comparing BV/TV between OVX and sham groups. ■, SMD of each study; horizontal lines represent the 95% CI for the data; ◆, combined overall effect. BV/TV, trabecular bone volume fraction; OVX, ovariectomy; SMD, standardized mean difference; SD, standard deviation; CI, confidence interval

Nine studies included BV/TV as an outcome. Meta-analysis using a random-effects model indicated that the mandibles of the OVX rats exhibited significantly lower BV/TV values (SMD = − 2.41, 95% CI: − 3.51 to − 1.31, *P* < 0.01, Fig. 2). High heterogeneity among the included studies was detected (Cochrane Q test: *P* < 0.01, I^2^ = 81%, Fig. 2).

#### Tb.Th changes in the mandibles of OVX rats

**Fig. 3:**
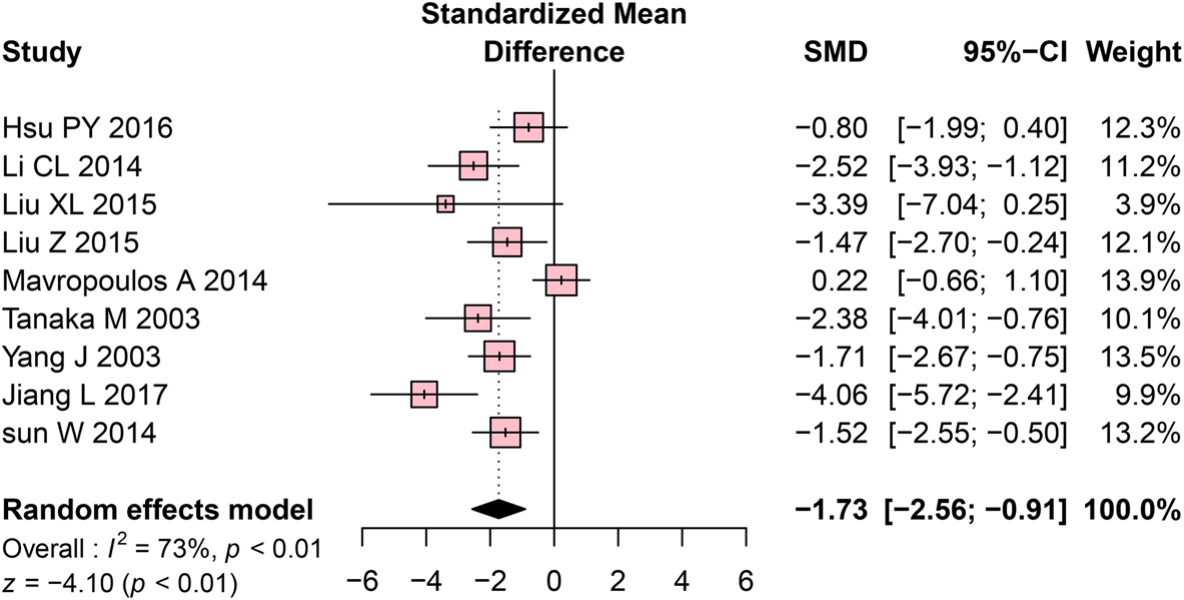
Forest plot comparing Tb.Th between OVX and sham groups. ■, SMD of each study; horizontal lines represent the 95% CI for the data; ◆, combined overall effect. Tb.Th, trabecular thickness; OVX, ovariectomy; SMD, standardized mean difference; SD, standard deviation; CI, confidence interval

Tb.Th was measured in 9 studies. The meta-analysis using a random-effects model revealed that the OVX group had significantly lower Tb.Th values than the control group (SMD = − 1.73, 95% CI: − 2.56 to − 0.91, *P* < 0.01, Fig. 3). High heterogeneity among the included studies was detected (Cochrane Q test: *P* < 0.01, I^2^ = 73%, Fig. 3).

#### Tb.Sp changes in the mandibles of OVX rats

**Fig. 4:**
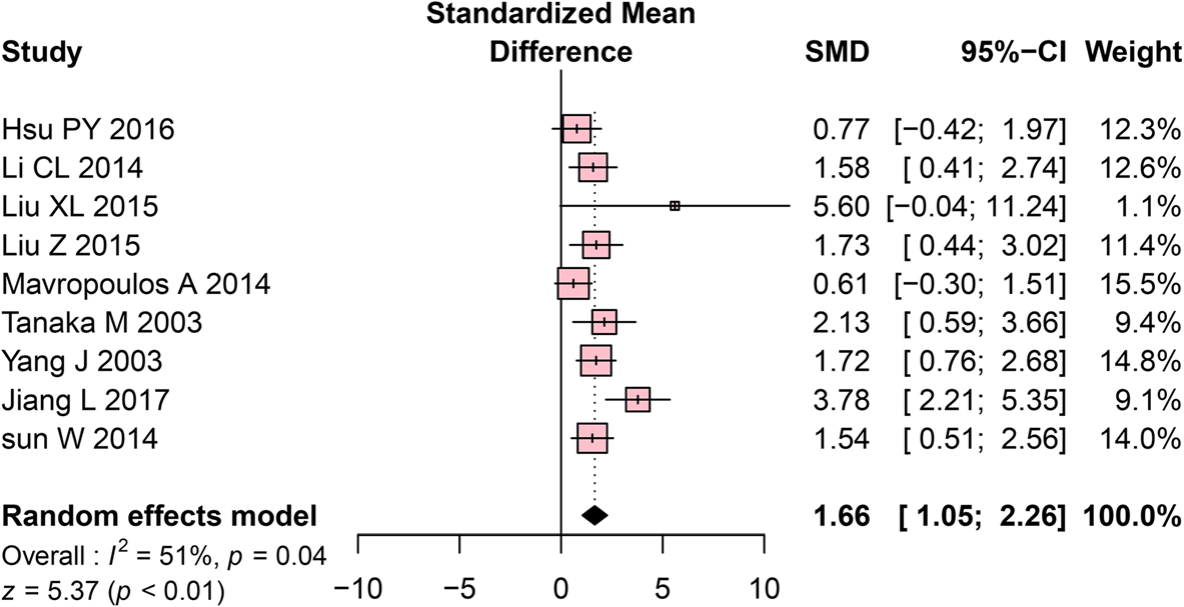
Forest plot comparing Tb.Sp between OVX and sham groups. ■, SMD of each study; horizontal lines represent the 95% CI for the data; ◆, combined overall effect. Tb. Sp, trabecular separation; OVX, ovariectomy; SMD, standardized mean difference; SD, standard deviation; CI, confidence interval

Nine studies assessed Tb.Sp. The meta-analysis using a random-effects model revealed that the OVX group has significantly higher Tb.Sp values than the control group (SMD = 1.66, 95% CI: 1.05 to 2.26, *P* < 0.01, Fig. 4). High heterogeneity among the included studies was detected (Cochrane Q test: *P* = 0.04, I^2^ = 51%, Fig. 4).

#### Tb.N changes in the mandibles of OVX rats

**Fig. 5:**
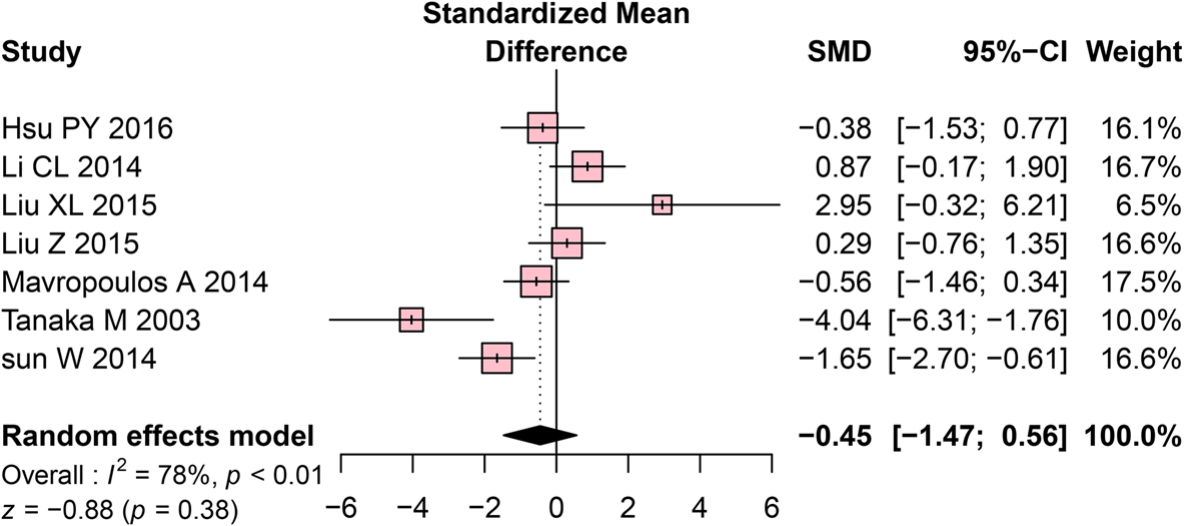
Forest plot comparing Tb. N between OVX and sham groups. ■, SMD of each study; horizontal lines represent the 95% CI for the data; ◆, combined overall effect. Tb.N, trabecular number; OVX, ovariectomy; SMD, standardized mean difference; SD, standard deviation; CI, confidence interval

Only 7 studies included measures of Tb.N, with a very small effect size (SMD = − 0.45, 95% CI: − 1.47 to 0.56); non-significant effects were found (*P* = 0.38, Fig. 5).

#### BMD changes in the mandibles of OVX rats

**Fig. 6:**
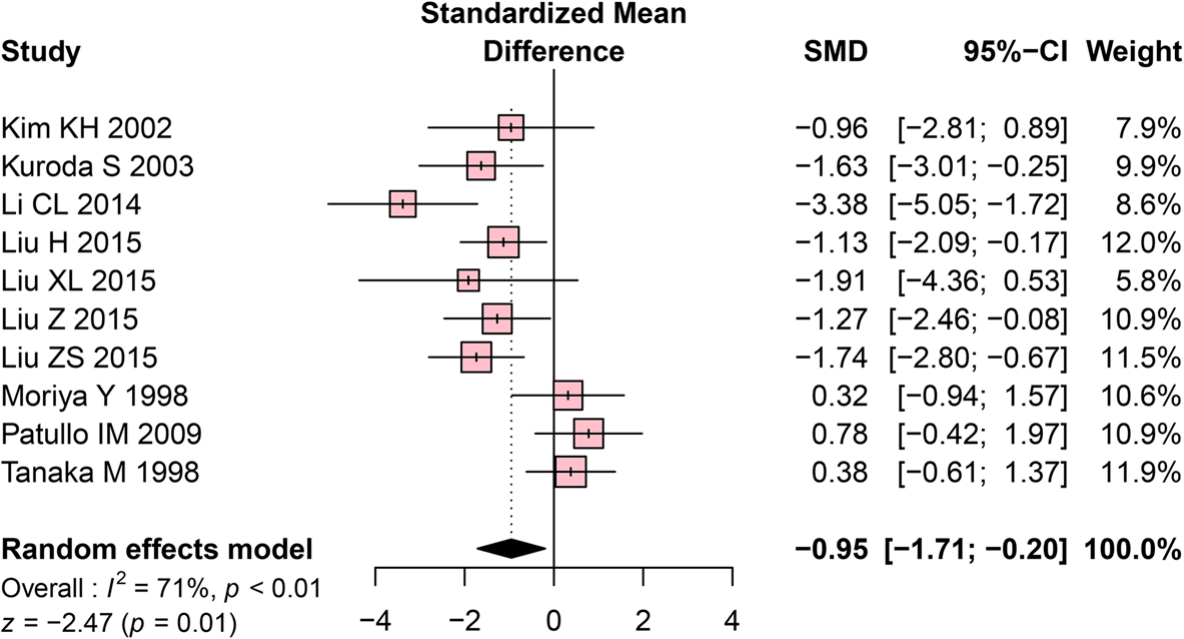
Forest plot comparing BMD between OVX and sham groups. ■, SMD of each study; horizontal lines represent the 95% CI for the data; ◆, combined overall effect. BMD, bone mineral density; OVX, ovariectomy; SMD, standardized mean difference; SD, standard deviation; CI, confidence interval

An analysis of BMD data comprising 12 intergroup comparisons, generated from 16 original studies, was performed. The meta-analysis using a random-effects model revealed that the OVX group had significantly lower BMD values than the control group (SMD = − 0.95, 95% CI: − 1.71 to − 0.20, *P* = 0.01, Fig. 6). High heterogeneity among the included studies was detected (Cochrane Q test: *P* < 0.001, I^2^ = 71%, Fig. 6).

### Subgroup analysis

To further explore the potential sources leading to heterogeneity, we performed a subgroup analysis based on strain, ROI and quality score. After stratification by strain, no subgroup differences were displayed. The comparison of the OVX and control group in Sprague-Dawley rats showed significant differences in BV/TV, Tb.Th, Tb.Sp, and BMD outcome. Sprague-Dawley rat group has sustained the high heterogeneity between the studies. In comparison, the subgroup of Wistar rats showed significant differences between the OVX and control groups in BV/TV, Tb.Th, and Tb.Sp and revealed a reduction in heterogeneity in parameters except for BMD.

Stratification by ROI led to a statistically significant result in subgroup differences among the mandibular body, mandibular condyle, and interradicular septum of the M1. The interradicular septum of the M1 subgroup has shown significant differences between the OVX and control groups in BV/TV, Tb.Th, Tb.Sp and BMD outcomes. There was a reduction of heterogeneity in interradicular septum of the M1 except for Tb.N. Moreover, the mandibular body subgroup showed significant differences between the OVX and control groups in BV/TV, Tb.Sp and Tb.N, with no heterogeneity among the studies.

The Quality score has been divided by high quality and low-quality subgroup, the high-quality has 4 scores out of 7, low-quality has 3 scores out of 7. The high quality and low quality have not shown subgroup difference. In a high-quality group, there was a significant difference between OVX and control groups in Tb.Th, Tb.Sp. The low-quality group, there was a significant difference between OVX and control groups in BT/TV, Tb.Sp. Except for low-quality studies, there is no effect on heterogeneity. The detailed results of the subgroup analysis are shown in Table 4.

**Table 4:** Results of the subgroup analysis of microarchitectural changes in the mandible of OVX rats

Moderator	Number of studies	SMD (95% CI)	I^2^	*P*-value of Z	Between-subgroup difference	
					Q statistic	*P*-value
BV/TV						
Strain						
Fischer	1	-4.00 (-6.25, -1.74)	-	-	0.92	0.82
Lewis-Brown-Norway	1	-1.48 (-2.40, -0.55)	-	-		
Sprague-Dawley	5	-2.75 (-4.84, -0.66)	89%	0.01		
Wistar	2	-1.92 (-2.92, -0.92)	0%	<0.001		
ROI						
Mandibular body	4	-1.12 (-2.10, -0.13)	72%	0.026	13.89	0.001
Mandibular condyle	1	-5.13 (-7.12, -3.15)	-	-		
M1 interradicular septum	4	-3.29 (-4.34, -2.25)	5%	<0.001		
Quality						
High	5	-3.19 (-4.62, -1.76)	74%	0.052	3.02	0.082
Low	4	-1.37 (-2.75, 0.01)	77%	<0.001		
Tb.Th						
Strain						
Fischer	1	-2.38 (-4.01, -0.76)	-	-	0.59	0.899
Lewis-Brown-Norway	1	-1.71 (-2.67, -0.75)	-	-		
Sprague-Dawley	5	-2.04 (-3.63, -0.43)	85%	0.013		
Wistar	2	-1.12 (-1.98, -0.23)	0%	0.01		
ROI						
Mandibular body	4	-0.94 (-1.88, 0.00)	71%	0.05	9.18	0.01
Mandibular condyle	1	-4.06 (-5.72, -2.41)	-	-		
M1 interradicular septum	4	-2.10 (-2.88, -1.32)	0%	<0.001		
Quality						
High	5	-2.15 (-3.16, -1.13)	65%	<0.001	1.47	0.226
Low	4	-1.18 (-2.44, 0.08)	74%	0.067		
Tb.Sp						
Strain						
Fischer	1	2.13 (0.59, 3.66)	-	-	0.55	0.908
Lewis-Brown-Norway	1	1.72 (0.76, 2.68)	-	-		
Sprague-Dawley	5	1.89 ( 0.75, 3.04)	71%	0.001		
Wistar	2	1.22 (0.28, 2.15)	12%	0.011		
ROI						
Mandibular body	4	1.17 (0.61, 1.73)	19%	<0.001	10.5	0.005
Mandibular condyle	1	3.78 (2.21, 5.35)	-	-		
M1 interradicular septum	4	1.83 (1.08, 2.58)	0%	<0.001		
Quality						
High	5	1.85 (0.98, 2.73)	58%	<0.001	0.39	0.533
Low	4	1.41 (0.51, 2.31)	47%	0.002		
Tb.N						
Strain						
Fischer	1	-4.04 (-6.31, -1.76)	-	-	5.92	0.051
Sprague-Dawley	4	-0.04 ( -1.45, 1.36)	80%	0.955		
Wistar	2	-0.02 (-0.79, 0.76)	0%	0.97		
ROI						
Mandibular body	3	-0.86 (-1.62, -0.10)	40%	0.190	0.57	0.451
M1 interradicular septum	4	-0.06 (-1.99, 1.87)	83%	0.845		
Quality						
High	4	-1.09 (-2.73, 0.55)	85%	0.193	1.58	0.209
Low	3	0.20 (-1.02, 1.43)	59%	0.744		
BMD						
Strain						
Fischer	1	0.38 (-0.61, 1.37)	-	-	1.97	0.373
Sprague-Dawley	6	-1.35 (-2.33, -0.37)	62%	0.007		
Wistar	3	-0.76 (-2.26, 0.74)	81%	0.323		
ROI						
Mandibular body	3	-0.60 (-1.56, 0.37)	40%	0.225	7.91	0.048
Mandibular condyle	2	-0.57 (-2.53, 1.40)	81%	0.572		
Entire mandible	1	-0.78 (-0.42, 1.97)	-	0.201		
M1 interradicular septum	4	-1.93 (-2.79, -1.08)	29%	<0.001		
Quality						
High	4	-0.95 (-2.67, 0.76)	86%	0.225	0.01	0.915
Low	6	-0.93 (-1.64, -0.22)	42%	0.572		

*OVX* ovariectomy, *SMD* standardized mean difference, *CI* confidence interval, *BV/TV* trabecular bone volume fraction, *Tb.Th* trabecular thickness, *Tb.Sp* trabecular separation, *Tb.N* trabecular number, *BMD* bone mineral density, *ROI* region of interest, *M1* first molar

### Meta-regression analysis

**Fig. 7:**
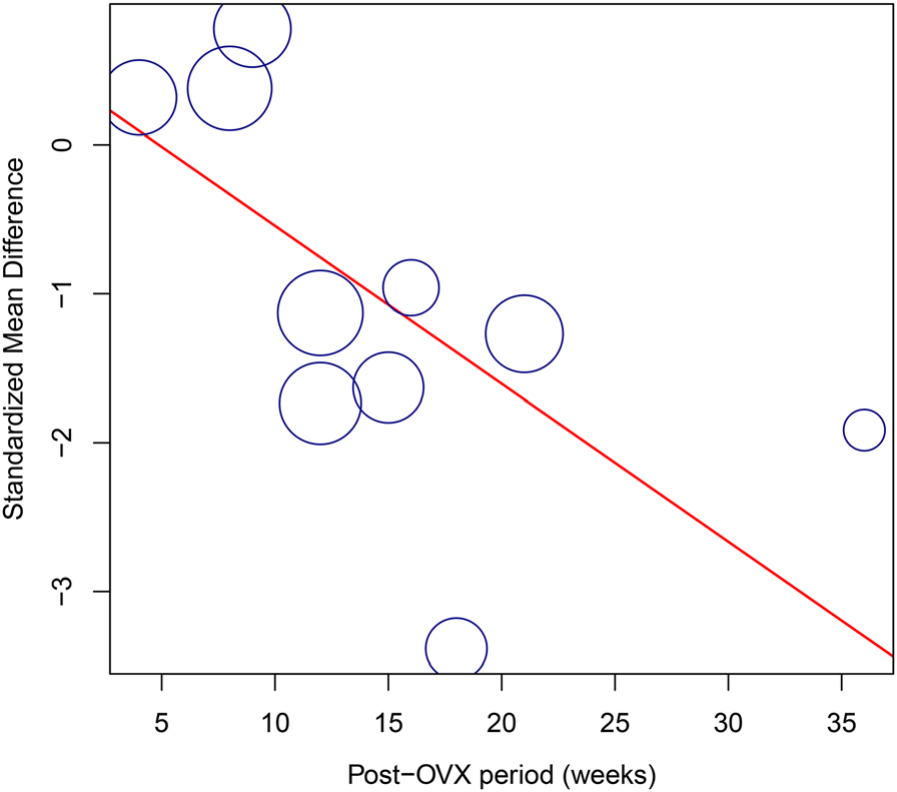
Meta-regression of the relationship between post-OVX periods and bone mineral density. Each circle represents the SMD of an individual study, with the circle size denoting the precision of the estimate. OVX, ovariectomy; SMD, standardized mean difference

The potential influence of the characteristics of continuous variables such as the rats’ age at OVX and the post-OVX period was assessed with meta-regression. We discovered that the duration after OVX was the factor with a significant influence on the heterogeneity of BMD in the meta-analysis (*β* = − 0.106, 95% CI: − 0.20 to − 0.02, *P* = 0.017). In the meta-regression plot, the duration after OVX showed an inverse relationship with SMD (Fig. 7). The rats’ age showed no statistically significant effects on bone microstructure outcomes. Detailed results of the meta-regression analysis are presented in Table 5.

**Table 5:** Results of the meta-regression analyses by rats’ age and post-OVX period

Dependent variable	Moderator	Number of studies	*β*	95% CI	*P*-value
BV/TV	Rats’ age	8	−0.049	−0.26, 0.16	0.641
	Post-OVX period	9	−0.048	−0.14, 0.05	0.319
Tb.Th	Rats’ age	8	−0.045	−0.20, 0.11	0.569
	Post-OVX period	9	−0.024	−0.10, 0.05	0.514
Tb.Sp	Rats’ age	8	0.055	−0.06, 0.17	0.342
	Post-OVX period	9	0.018	−0.04, 0.08	0.532
Tb.N	Rats’ age	6	−0.068	−0.22, 0.09	0.387
	Post-OVX period	7	0.086	−0.05, 0.22	0.212
BMD	Rats’ age	9	−0.004	−0.16, 0.15	0.958
	Post-OVX period	10	−0.106	−0.20, − 0.02	0.017

*OVX* Ovariectomy, *BV/TV* Trabecular bone volume fraction, *Tb.Th* Trabecular thickness, *Tb. Sp* Trabecular separation, *Tb.N* Trabecular number, *BMD* Bone mineral density, *CI* Confidence interval

### Sensitivity analysis

We conducted a sensitivity analysis to assess the robustness of our results. For studies of OVX rat on BMD, exclusion of the shorter post-OVX period sample [15, 19, 34, 36, 37] considerably enhanced the effect size (SMD = − 1.77, 95% CI: − 2.57 to − 0.96, *P* < 0.001). The heterogeneity (I^2^) among the studies was reduced from 71 to 21%.

In studies using the mandibular body with ROI, exclusion of the shorter post-OVX period sample considerably increased the effect size of BMD (SMD = − 1.09, 95% CI: − 1.95 to − 0.24, *P* = 0.012) and there was no heterogeneity among the studies (I^2^ = 0%, *P* = 0.873). For OVX rat using M1 interradicular septum, except for the shorter post-OVX period studies, increased the effect size of BMD (SMD = − 2.13, 95% CI: − 3.52 to − 0.73, *P* = 0.003) with slightly increased heterogeneity (I^2^ = 51%, *P* = 0.129).

### Publication bias

**Fig. 8:**
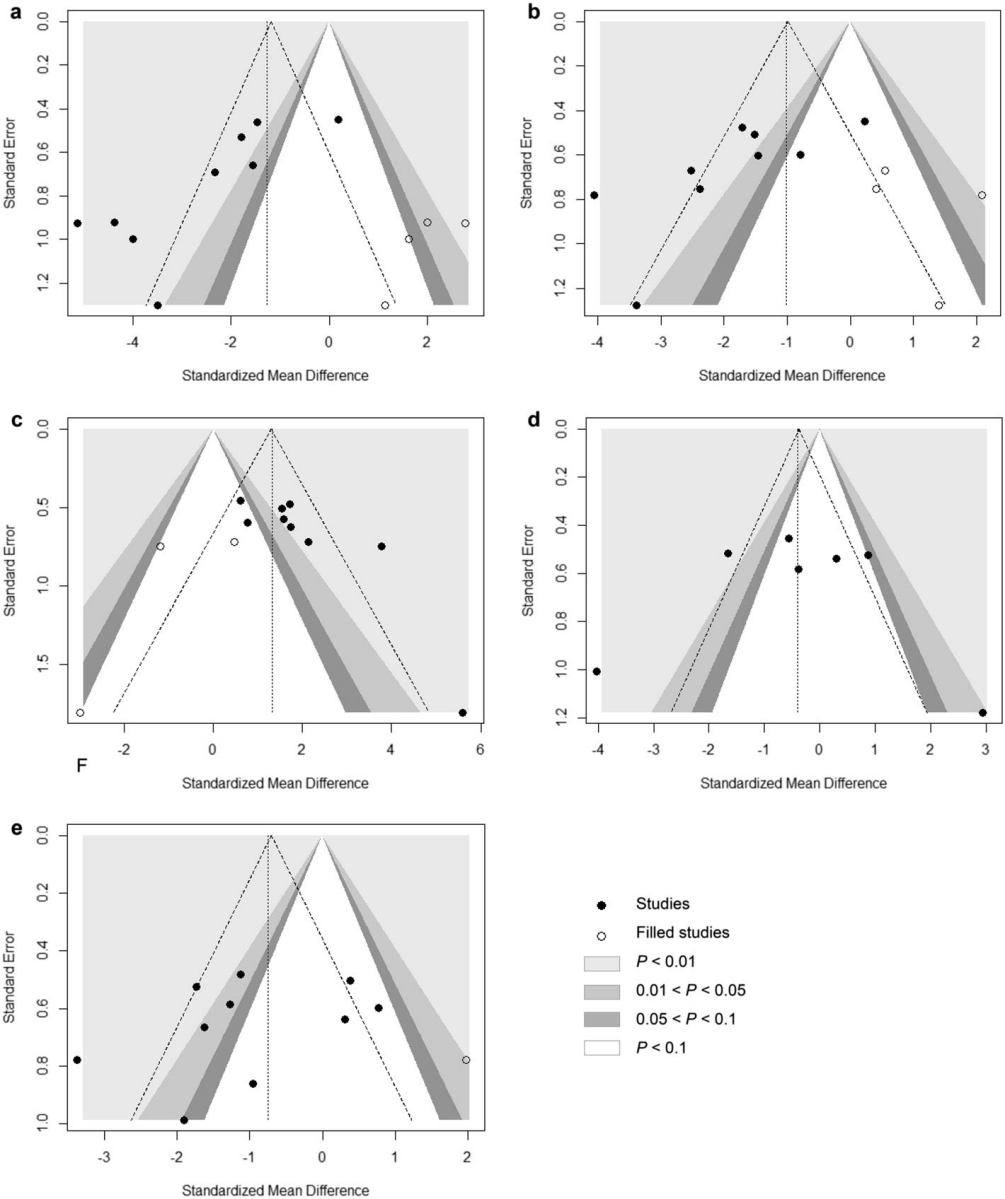
Contour-enhanced funnel plot for the analysis of publication bias, combined with a trim-and-fill analysis. **a** Funnel plot of publication bias in BV/TV changes in the OVX rats. **b** Funnel plot of publication bias in Tb.Th changes in the OVX rats. **c** Funnel plot of publication bias in Tb.Sp changes in the OVX rats. **d** Funnel plot of publication bias in Tb. N changes in the OVX rats. **e** Funnel plot of publication bias in BMD changes in the OVX rats. OVX, ovariectomy; BV/TV, trabecular bone volume fraction; Tb.Th, trabecular number; Tb. Sp, trabecular thickness; Tb.N, trabecular separation; BMD, bone mineral density

The Begg’s funnel plot and the Egger’s test were used to assessing the publication bias of the studies. Contour-enhanced funnel plots revealed no evidence of publication bias for Tb. N or BMD (Fig. 8d and e), as confirmed by the Begg’s test and Egger test (Tb.N: *P*_*Begg*_ = 1.00, *P*_*Egger*_ = 0.386; BMD: *P*_*Begg*_ = 0.484, *P*_*Egger*_ = 0.214, Table 6). However, asymmetries were found in the contour-enhanced funnel plots for BV/TV, Tb.Th, and Tb.Sp indicating the presence of publication bias (Fig. 8a, b, and c). Moreover, the results of the Begg and Egger’s tests also provided clear evidence of publication bias (BV/TV: *P*_*Begg*_ = 0.012, *P*_*Egger*_ = 0.005; Tb.Th: *P*_*Begg*_ = 0.037, *P*_*Egger*_ = 0.034; Tb.Sp: *P*_*Begg*_ = 0.022, *P*_*Egger*_ = 0.022; Table 6).

**Table 6:** Publication bias assessment in the meta-analysis of microarchitectural changes in the mandibles of OVX rats

	Begg’s test *P* value	Egger’s test *P* value	Fail-safe N	Trim-and-fill	
				Adjusted SMD (95% CI)	*P-*value
BV/TV	0.012	0.005	300	−1.27 (−2.42, −1.11)	0.032
Tb.Th	0.037	0.034	215	−1.01 (− 1.87, − 0.16)	0.020
Tb.Sp	0.022	0.022	239	1.31 (0.57, 2.05)	< 0.001
Tb.N	1.00	0.386	3	−0.47 (−1.48, 0.53)	0.354
BMD	0.484	0.214	75	−0.75 (−1.56, 0.06)	0.070

*OVX* Ovariectomy, *BV/TV* Trabecular bone volume fraction, *Tb.Th* Trabecular number, *Tb. Sp* Trabecular thickness, *Tb.N* Trabecular separation, *BMD* Bone mineral density, *SMD* Standardized mean difference

The trim-and-fill method was implemented to estimate the influence of publication bias by imputing potentially missing studies. After adjusting for publication bias, the effect size of these 3 outcomes was reduced, yet they remained statistically significant. (BV/TV: *P* = 0.032, Tb.Th: *P* = 0.020, Tb.Sp: *P* < 0.001). Additionally, a high fail-safe number was found for each outcome (300 for BV/TV, 215 for Tb.Th, and 239 for Tb.Sp). This number represents the minimum number of unpublished studies required to make the meta-analysis non-significant. The full details of the publication bias analysis are shown in Table 6.

## Discussion

Heterogeneity in animal studies is caused by variation in research methods; biological characteristics based on species, sex, and age; interventions; and measurements of the main effect [18, 41]. This meta-analysis of systemic osteoporosis and mandibular bone changes showed that the outcomes were heterogeneous across studies. Investigating the causes and effects of this heterogeneity can assist in designing experiments to maximize the usefulness of animal models.

BMD is commonly used in clinical analysis as a valuable tool for osteoporosis risk assessment [42]. Bone morphometric parameters such as BV/TV, Tb.Th, Tb.Sp and Tb. N are also commonly measured on micro-CT images to assess the quality of the bone [43]. This meta-analysis revealed radiologic microarchitectural change consistent with osteoporosis in the mandibles of OVX rats. BV/TV, Tb.Th, Tb.Sp and BMD displayed consistent with bone loss, in terms of the effect size, in the OVX group compared to control groups, while Tb. N did not show a significant bone loss. This result implies that Tb. N is an ambiguous parameter for detecting bone microstructure changes in the mandible. The Tb. N implicates average of the trabecular number which requires a long-term observation until the number of trabecular changes. Nonetheless, the thickness and separation denote the change of microstructure in a predictable time period to produce the matured outcome.

The meta-analysis performed in this study found heterogeneity across studies for all outcomes (BV/TV, Tb.Th, Tb.Sp, Tb.N, and BMD). The impact of various characteristics was investigated through a subgroup analysis, meta-regression, and sensitivity analysis.

The trabecular bone in the femur and lumbar vertebrae has generally been analyzed with an ROI located 1 mm above the epiphyseal growth plate [44]. However, it is difficult to establish a unified ROI for the mandible due to the presence of teeth and the complex morphology of the mandible, which consists of the alveolar process, basal bone, and condyle [45]. In this meta-analysis, the mandibular body, condyle, M1 interradicular septum, and/or the entire mandible were selected as the ROI. In the subgroup analysis, the mandibular ROI was found to be an influential moderator of microarchitectural changes in response to estrogen deficiency. As Johnston and Ward [46] suggested, the M1 interradicular septum should be used as the ROI in the mandible due to its well-characterized site and distinctive response to estrogen depletion [46]. Thus, the molar region of the mandible is a definable model that has been widely used [47]. Similarly, the results of our subgroup analysis reflect significant differences in the M1 interradicular septum, showing robust bone microstructural changes between the OVX and control groups.

OVX-induced alterations in the proximal tibia, the femoral neck, and lumbar spine are known to reduce trabecular bone volume in fewer than 12 weeks after OVX [48–51]. However, the post-OVX period required for bone structural changes to occur in the mandible is not conclusively known. The majority of studies had cost and time restraints which restricts a single time period was observed for bone deterioration in the mandible based on the effects of osteoporosis. In our quantitative meta-analysis, meta-regression indicated an association between the post-OVX period and BMD changes in the rats’ mandible. This result reported that with longer post-OVX periods, a more notable decrease in BMD was exhibited. In addition, the sensitivity analysis revealed that the heterogeneity among the studies of BMD in OVX rats was reduced by excluding samples with a shorter post-OVX period. As the osteoporosis review article by Dervis [52] argued, insufficient duration of the experimental investigation leads to a failure to detect OVX-induced changes in the mandible. Johnston and Ward’s [46] review states that longer post-OVX periods are associated with greater effects on bone structural changes in the mandible. Therefore, a sufficient OVX duration should be considered as part of the design of studies using OVX rats.

The most common types of osteoporosis rat models are sexually mature models (3 months old) and skeletally mature models (12 months old) [8]. The notion of using skeletally mature animals is based on their similarity to human cases of postmenopausal bone loss [53]. In contrast, sexually mature models are used to eliminate the possibility of bone loss and disease caused by aging [8]. Most of the studies included chose 12-week-old rats as sexually mature rats, and rats aged 24 weeks as skeletally immature. However, 3 of the studies used rats under 8 weeks, which is known to be inappropriate from Kalu’s [8] review, which found that young rats may have lower bone mass due to repressed growth in contrast to control groups with rapid growth. Thus, bone loss in rats under 8 weeks may result from impaired growth, rather than accelerated bone loss, as observed following menopause. The exploration of heterogeneity among studies showed that the age of the rats did not affect heterogeneity. Our meta-regression results suggest that differences in the rats’ age at OVX had no effect on OVX-induced changes in the mandible. Therefore, our results concur with those of a previous study that reported that the confounding effect on bone loss was greatly reduced as rat growth slowed after 12 weeks of age [53]. Although skeletal growth is not complete, 12-week-old OVX rat models are most commonly used for osteoporosis modeling because they have similar characteristics to skeletally mature rats [54]. In addition, sexually mature rats respond much more rapidly to OVX than aged rats, reducing the time and cost of the study [53].

Publication bias is a considerable problem for the validity of meta-analyses, as studies with significant or positive results have a better chance to be published than studies with non-significant results [55]. Systematic reviews of animal studies are more vulnerable to publication bias than those of clinical trials [56]. Non-significant or unfavorable studies may not be published for commercial reasons [38]. This meta-analysis identified clear publication bias for the outcomes of BV/TV, Tb.Th, and Tb.Sp. However, the trim-and-fill method indicated that the publication bias led to minor changes in the effect size and demonstrated a high fail-safe number. Based on this result, our meta-analysis can be considered reliable and not meaningfully influenced by publication bias.

The results of the meta-analysis must be interpreted cautiously in accordance with the limitations of this study. As only full-text articles in English and Korean were included in the meta-analysis, eligible studies in other languages may have been overlooked. The absence of clarification about the random allocation method and lack of blind assessments reflect low methodological quality in the included studies. The statistical power may not have been sufficient, as a limited number of studies were included in the subgroup analysis and meta-regression. However, the findings of this study can fully assist in the design and interpretation of further studies and in the selection of an OVX animal model.

## Conclusions

In conclusion, our study indicates that the mandibular microarchitectural changes and OVX-induced osteoporosis in the rat model show a significant relationship. However, heterogeneity showed that the differences in the ROI and post-OVX period had an effect on bone microstructural changes in OVX rats. Based on our heterogeneity assessment, the following experimental design for micro CT studies of mandibular osteoporosis in a rat model can be proposed: a 12 week-old rat model, use of the M1 interradicular septum as the ROI, and a sufficient observation period after ovariectomy.

## Data Availability

All data generated or analyzed during this study are included within the article.

## Abbreviations

BMD: Bone mineral density
BV/TV: Trabecular bone volume fraction
CIs: Confidence intervals
M1: First molar
Micro-CT: Micro-computed tomography
OVX: Ovariectomized
PRISMA: Preferred Reporting Items for Systematic Review and Meta-analysis
ROI: Region of interest
SMD: Standardized mean difference
Tb.N: Trabecular number
Tb.Sp: Trabecular separation
Tb.Th: Trabecular thickness
